# *Daphne mucronata* enhances cell proliferation and protects human adipose stem cells against monosodium iodoacetate induced oxidative stress *in vitro*

**DOI:** 10.1080/21623945.2020.1812242

**Published:** 2020-08-31

**Authors:** Numan Fazal, Hamzah Khawaja, Nadia Naseer, Azim Jahangir Khan, Noreen Latief

**Affiliations:** a National Centre of Excellence in Molecular Biology, University of the Punjab, Lahore, Pakistan; b Allama Iqbal Medical College, University of Health Sciences, Lahore, Pakistan

**Keywords:** Mesenchymal stem cells, *Daphne mucronata*, preconditioning, cell viability, proliferation, monosodium iodoacetate

## Abstract

Mesenchymal stem cells (MSCs) are being used to treat many diseases as they exhibit great regenerative potential. However, MSC’s transplantation sometimes does not yield the maximum regenerative outcome as they are unable to survive in inflammatory conditions. Several approaches including preconditioning are used to improve the survival rate of mesenchymal stem cells. One such recently reported approach is preconditioning MSCs with plant extracts. The present study was designed to evaluate the effect of *Daphne mucronata* extract on stressed human adipose-derived mesenchymal stem cells (hADMSCs). Isolated hADMSCs were preconditioned with different concentrations of *Daphne muconata* extract and the protective, proliferative, antioxidant and anti-inflammatory effect was assessed through various assays and expression analysis of inflammatory markers regulated through NF-κB pathway. Results suggest that preconditioning hADMSCs with *Daphne mucronata* increased the cell viability, proliferative and protective potential of hADMSCs with a concomitant reduction in LDH, ROS and elevation in SOD activity. Moreover, both the ELISA and gene expression analysis demonstrated down regulations of inflammatory markers (IL1-β, TNF-α, p65, p50, MMP13) in *Daphne mucronata* preconditioned hADMSCs as compared to stress. This is the first study to report the use of MIA induced oxidative stress against hADMSC’s and effect of *Daphne mucronata* on stressed hADMSCs. Results of these studies provided evidence that *Daphne mucronata* protects the hADMSCs during stress conditions by down regulating the inflammatory markers and hence increase the viability and proliferative potential of hADMSCs that is crucial for transplantation purposes.

## Graphical abstract

**Figure:**
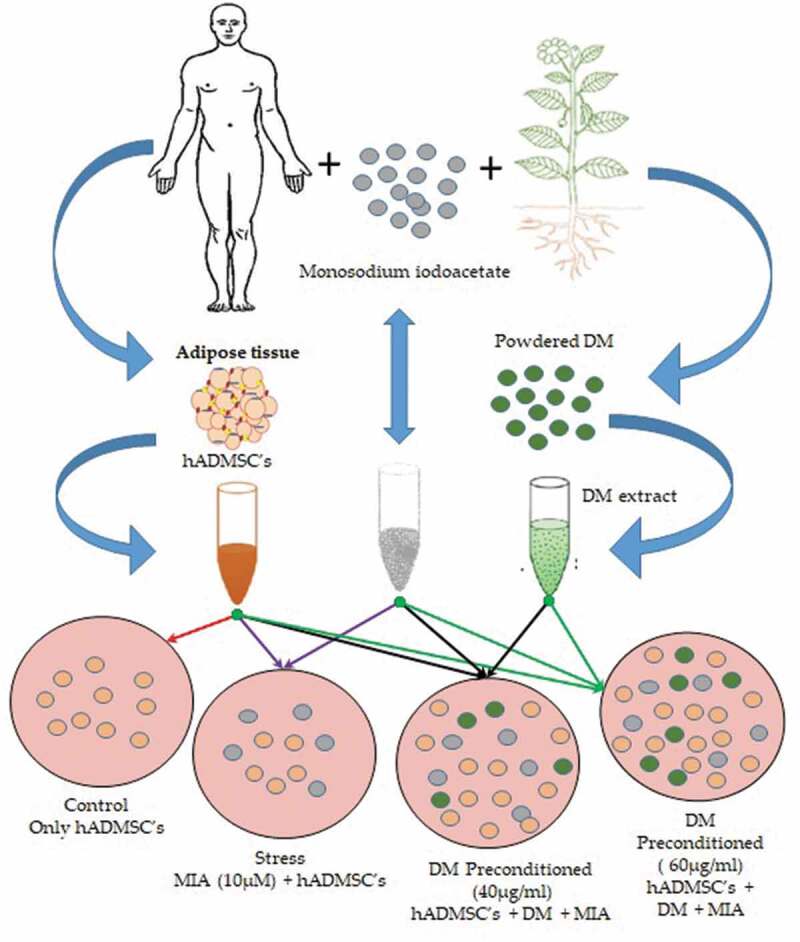

## 1. Introduction

Recently, scientists and clinicians have put their efforts in exploring Mesenchymal stem cells (MSCs) because of their inherent potential of multilineage differentiation, tissue repair and regeneration after migration to the site of tissue injury [1–3]. MSCs can be isolated from various human sources including adipose tissue [4–6] skeletal muscle [7] umbilical cord blood and Wharton’s jelly [7–10]. According to the international Society for Cellular Therapy (ISCT), MSCs must be positive for CD73, CD90,and CD105 and negative for CD11b or CD14, CD19 orCD79a, CD34, CD45 [11]. MSCs induced by proper signals can be differentiated into a large number of specific cell types including osteoblasts, adipocytes, chondrocytes, cardiomyocytes and endothelial cells [12–14]. Despite a large number of published work regarding the benefits of MSCs in preclinical experimental settings, the use of MSCs in treating patients with various immune diseases showed variable results [15,16]. The utility of MSCs is restricted due to pathophysiological environmental conditions, such as oxidative stress, low oxygen levels, limited nutrient supply and inflammation [17]. To produce functional MSCs with high therapeutic efficiency, protection against several stressors and optimization of MSC’s culture conditions are required. We believe that preconditioning, hypoxic culture, pretreatment and genetic manipulation will increase the survival of MSC’s [17]. One approach to improve the ability of MSCs to survive in the harsh microenvironment is to precondition the cells *ex vivo* in the specifically engineered environment with different physical or chemical agents [18].

The protective and therapeutic effect of various phytochemicals has been evaluated in human mesenchymal stem cells [19–21]. Furthermore, several medicinal plants extract have been reported to have anti-inflammatory effect by inhibiting inflammatory pathways including NF-kB pathway [22,23]. It may be realized that NF-κB is a multi-functional transcription factor that is involved in various biological processes including inflammation, apoptosis, and immune regulation [24]. NF-κB induces the expression of various pro-inflammatory genes, including those encoding cytokines and chemokines [25].

Preconditioned MSCs exhibit increased survival, proliferation as well as therapeutic potential because they can better cope with inflammatory conditions both *in vitro* and *in vivo* as compared to the non-preconditioned MSCs [26].

Various stressors including cobalt chloride, hydrogen peroxide and Lipopolysaccharide are used to induce oxidative stress *in vitro* [27–29]. Monosodium iodoacetate (MIA) is usually used to induce stress in chondrocytes both *in vitro* and *in vivo* [30–34]. MIA promotes ROS level thereby causing membrane potential alterations, upregulates caspase-3 activity and enhances the release of cytochrome c that ultimately leading chondrocyte to apoptosis [35]. Herein, we assessed the production of ROS in hADMSCs through assays and its effect was evaluated at gene and protein level.

*Daphne mucronata* (DM), a shrub abundantly found in different regions of Asia and is commonly used as folk medicine in Iran and northern areas of Pakistan [36]. Traditionally, it is used to treat musculoskeletal problems [37,38]. DM is also reported to exhibit anti-inflammatory activity against various cell lines [39]. However, its protective effect against MIA induced oxidative stress in hADMSCs is not yet studied. The present work reports the protective, proliferative, anti-oxidant and anti-inflammatory effect of *Daphne mucronata* extract against Monosodium iodoacetate induced stress by downregulating NF-κB and associated inflammatory markers in hADMSCs *in vitro*. This study also confirms the first time use of MIA induced oxidative stress against hADMSCs *in vitro*.

## 2. Materials and methods

### 2.1 Plant collection and extract preparation

Fresh leaves of *Daphne mucronata* (DM) plants were collected from district Swat, Khyber Pukhtunkhwa, Pakistan. The specimen (Voucher # LAH35847) was submitted to the herbarium of University of the Punjab, Lahore, Pakistan. Collected leaves were first shade dried at room temperature and then ground into a fine powder. The fine powdered material was weighted and 100 g of the powder was suspended in 3× methanol and placed for 2 days in the shaker (New Brunswick™ Innova® 44/44 R) and filtered through Whatmann filter paper. The residue remained was again suspended in 2× methanol and placed on shaker for 2 days and filtered through Whatmann filter paper. The residue remained was finally suspended in 1× methanol, placed on shaker for 1 day and filtered. The filtrate was pooled and evaporated to dryness in an incubator at 37°C for few days to obtain final extract.

### 2.2 Phytochemical analysis and antioxidant determination

The plant extract was further evaluated for phytochemical analysis to determine the total phenolic content (TPC), total flavonoid content (TFC) and antioxidant potential through DPPH assay. Aluminium chloride colorimetric method and Folin–Ciocalteu reagent was used to determine the total flavonoid content and total phenolic contents in DM extract with slight modification [40,41]. Quercetin calibration curve was used to calculate the TFC while Gallic acid calibration curve was used to estimate TPC in the extract expressed as μg per equivalent mg of dry weight. To determine the free radical scavenging estimation of DM extract, DPPH (2,2-diphenyl-1-picryl-hydrazyl-hydrate) technique was used [42]. The following formula was used to calculate the percentage (%) of free radical scavenging activity of the extract as:

DPPHfreeradicalscavengingeffect= [(Absorbanceofcontrol)−(Absorbanceofsample) ×100]/(Absorbanceofcontrol)

### 2.3 Adipose tissue culturing and immunophenotyping of isolated hADMSCs

Adipose stem cells were isolated from adipose tissue collected from hospital with the consent of patient (n = 3) going through liposuction. Isolation of adipose stem cells was performed following a previous protocol with slight modifications [43]. Briefly, the collected sample was washed thrice with 1× PBS. Washed adipose was treated with Collagenase 1A and incubated at 37°C for 45 minutes. Digested adipose was first filtered through a 100 µm mesh, treated with active media and then centrifuged for 10 minutes at 1200×*g*. SVF obtained in the pallet form was resuspended in 1 ml low-glucose Dulbecco’s modified eagle’s medium (LG-DMEM) and shifted to a 75cm^2^ flasks. Cells from passage 2 were further seeded for experimental purposes. Moreover, passage 2 cell were also subjected to Immunophenotyping through flow cytometery for CD 90-PE, CD73-PE, CD49D-PE, CD45-FITC, CD34-PE, CD106-FITC (BD Biosciences, USA) and CD105 (Santa Cruz Biotechnology, USA) according to minimal criteria of ISCT mentioned before for defining MSCs [11]. FACS were performed according to the already published protocol [44]

### 2.4 DM extract effect on metabolic activity and viability of ADMSCs

DM extract was first evaluated for its metabolic activity and viability against hADMSCs. Metabolic activity of hADMSCs was performed through MTT while the viability was checked through trypan blue exclusion assay. For MTT assay, 8000 cells/well from passage 2 were seeded using a 96 well culture plate and then preconditioned with different concentrations (1 µg/ml, 5 µg/ml, 10 µg/ml, 20 µg/ml, 40 µg/ml, 60 µg/ml) of DM extract for 24 hours. Cell metabolic activity was measured after 24 hours of incubation through microplate reader at 570 nm. Similarly, for trypan blue exclusion assay, preconditioned cells were trypsinized and resuspended in 1 ml of LG-DMEM culture media. 10 µl of the suspended cells were mixed with 10 ul of 0.4% trypan blue. Live and dead cells were counted under the microscope. Two concentrations (40 µg/ml, 60 µg/ml) were further selected to evaluate their preconditioning potential to cope with MIA stress.

### 2.5 Stress optimization, metabolic activity and viability of stressed ADMSCs after preconditioning

Stress was induced in hADMSCs using different monosodium iodoacetate concentrations (1 µM, 5 µM, 10 µM, 15 µM, 20 µM). Briefly, cells from passage 2 (8×10^3^ cells/well) were seeded in a 96 well plate. After attachment, cells were incubating with LG-DMEM media with different concentrations of MIA (1 µm, 5 µm, 8 µm, 10 µm, 15 µm, 20 µm) for 24 hours. Media was discarded after 24 hours and cells were analysed for their metabolic activity and viability.

Furthermore, to assess the preconditioning potential of DM extract, passage 2 cells (1×10^6^ cells/well) were seeded in a 96 well plate. Cells were then preconditioned with two different concentrations (40 µg/ml, 60 µg/ml) of DM extract and incubated for 24 hours. After incubation, media was removed and MIA stress (10 µM) was induced in cells for another 24 hrs. Metabolic activity and viability of cells were then evaluated using MTT and Trypan blue exclusion assay as described in section (2.4).

### 2.6 Wound healing assay

The migration potential of DM extract on hADMSCs was assessed through cell scratch assay. Briefly, 8 × 10^4^ cells were seeded in a 6-well plate and left to form a monolayer. The monolayer was then scratched using a 200-ul sterile pipette after the cells attained 80% confluency. Cells were then washed to remove non-adherent cells and medium was replaced. Images were taken using a phase contrast microscope soon after scratch creation followed by microscopy at Day1, Day 2 and Day 3. Furthermore, wound healing was assessed through ‘imagej’ software by comparing wound closure after Day1, Day 2 and Day 3 between control, DM 40 µg/ml and 60 µg/ml. We observed an overall 32.277% wound closure in control at day 3 while a percent wound closure of 40.776% and 46.091% was observed in DM 40 µg/ml and 60 µg/ml respectively.

### 2.7 Biochemical assays

Media from control, stress and DM preconditioned groups was collected and screened for levels of LDH (Roche Diagnostics, Cat No. 04744926001), superoxide dismutase (Abcam USA, Cat No. ab65354) and Reactive oxygen species (ROS) (Abcam, ab113851DCFDA) to assess the effect of DM on injured hADMSCs.

#### 2.7.1 Cytotoxicity assessment through LDH assay

LDH activity was measured in the media samples according to the manufacturer’s instructions (Roche Diagnostics, Cat No. 04744926001). Briefly, lactate dehydrogenase mixture was prepared by mixing equal volume of LDH co-factor, LDH dye solution and LDH substrate solution. About 50 μl of media samples were dispensed in 96-well plate and 100 µl of LDH assay mixture was added to each well. The plate was incubated for 20–30 minutes at room temperature. The reaction was stopped by the addition of stop solution and absorbance was measured through a microtiter plate reader at 490 nm and the reference absorbance at 690 nm.

#### 2.7.2 Superoxide dismutase (SOD) assay

SOD activity was determined using SOD assay kit (Abcam USA, Cat No. ab65354) according to the manufacturer’s instruction. 20 µl/well supernatant of different groups in triplicate was added followed by the addition of 200 µl of WST solution in each well included blank 1, 2 and 3. Then, added 20 µl of dilution buffer in blank 2 and 3. Enzyme working solution (20 µl) was added to each sample well and blank 1. Gently shake the plate for thoroughly mixing and incubated for 37°C for 20 minutes. Absorbance was measured at 450 nm using a microtiter plate reader.

Following equation was used for calculating SOD activity (% inhibition rate)

#### 2.7.3 Determination of ROS generated by hADMSCs

ROS assay is used to determine the quantity of reactive oxygen species produced by cells. Increase in ROS production starts decreasing the cells capability to ameliorate oxidative stress damage. ROS activity was measured by using Cellular ROS Detection Assay Kit, (Abcam, ab113851DCFDA) using the manufacturer’s protocol. Briefly, hADMSCs at a density of 25 × 104 were plated in a 96-well plate at p3 stage. Afterwards, cells were washed with 1× PBS three times and stained by 100 µl of DCF-DA working solution for 1hour at 37oC. The DCF-DA media was removed and cells were washed with 1× PBS. 100 μl/well PBS was added and fluorescence readings were taken immediately via spectrophotometer at 485 nm and 535 nm.

### 2.8 Semi-quantitative real-time polymerase chain reaction (PCR)

Total RNA from control, stress and preconditioned groups was isolated using trizol method. Isolated RNA (1 µg) was reverse transcribed into cDNA using cDNA synthesis kit (Thermo Scientific, Cat No: K1622). Real-time PCR was performed for different primers using Maxima SYBR Green/ROX qPCR Master Mix (2×) (Thermofisher cat # K0221). Sequences of the primers are shown in Table 1. All real-time PCR experiments were run in triplicate and mRNA levels of GAPDH were determined for the normalization of the IL1β, MMP13, P65, p50 and TNF-α mRNA expression values.

**Table 1.** List of primers used in the present study

S.NO	Gene		Primer Sequence (5՛ – -3՛)	Annealing temperature	Product size
1	IL1 β	Forward	GGCATCCAGCTACGAATCTC	59ᴼC	150
		Reverse	GAAGGGAAAGAAGGTGCTCA		
2	TNF-α	Forward	AACCTCCTCTCTGCCATCAA	60ᴼC	185
		Reverse	CCAAAGTAGACCTGCCCAGA		
3	RELA (p65)	Forward	CCCCAACTTTGTGGATGTCT	59ᴼC	475
		Reverse	CAGTGCTGTTGCACTGGTTT		
4	NFκB1 (p50)	Forward	GCGTTGTCCACAAGACAGAA	59ᴼC	176
		Reverse	CAGCCAGTGTTGTGATTGCT		
5	MMP-13	Forward	CGGGAATCCTGAAGGAGAAT	59ᴼC	152
		Reverse	TTCACCCACATCAGGAACCC		

### 2.9 Cytokines concentrations measurement through ELISA

Control, stress and DM preconditioned culture media was collected and stored at −40°C before use. NF-κB (P65) transcription factor ELISA (Abcam cat # ab133112) was performed on nuclear extracts instead of media. Collected media was used in triplicate. Cytokine concentrations were measured to further confirm the protective potential of DM extract. Collected media was subjected to ELISA for IL1β (Abcam cat # ab46052), TNF-α (Abcam cat # ab181421), IKKα and IKKβ (Cytoglow cat # CB5358) according to manufacturer’s protocol.

### 2.10 Statistical analysis

Statistical analysis of all results were presented as mean ± SD. Significant differences between the groups were determined by using one way ANOVA with Bonferroni’s test. A ‘p’ value less than 0.05 was considered significant statistically. Graphs were made through Graph-pad prism software (version 5.00 for Windows, GraphPad Software, USA). Moreover, all the experiments were run in triplicates (n = 3).

## 3. Results

### 3.1 Immunophenotyping of hADMSCs

**Figure 1.**
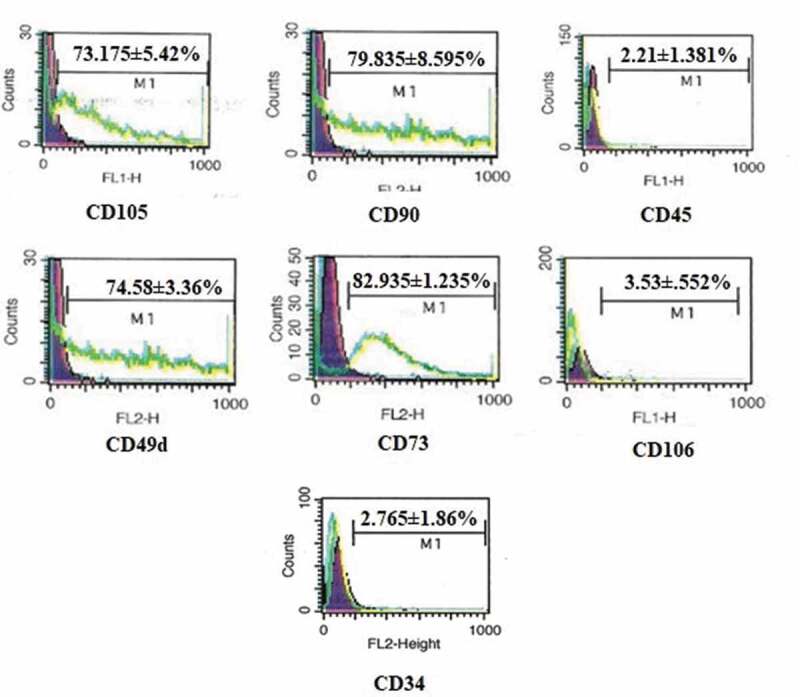
FACS analysis of hADMSCs for expression of CD105, CD90, CD73, CD45, CD106, CD49d and CD34 cell surface markers

Flow cytometry analysis of hADMSCs revealed the MSCs characterization of cells. The cultured hADMSCs showed positive expression of mesenchymal markers CD105 (73.175 ± 5.42%, CD73 (82.935 ± 1.235%), CD90 (79.835 ± 8.595%) and CD49d (74.58 ± 3.36%) while cells were negative for CD45 (2.21 ± 1.381%), CD34 (2.765 ± 1.86) and CD106 (3.53 ± 0.552%) as depicted in Figure 1.

### 3.2 DM extract exhibited phenolic and flavonoids content

DM extract showed high TPC and TFC content that ultimately resulted in a better scavenging activity when further subjected to DPPH assay. The phenolic and flavonoid content acts as reactive species scavengers and hence help in stabilizing the cells experiencing stress due to excess of reactive oxygen species. The total phenolic content of DM extract was 910 mg GAE/g while the total flavonoid content was found to be 50 mg QE/g. Furthermore, the DM extract has dark green colour, chemical odour and sticky by nature. A total of 6.7 g DM extract quantity was recovered from 100 g of initial powder extract with a percent yield of 6.7%.

### 3.3 Free radical scavenging capacity of DM extract

DPPH free radical scavenging assay was performed to determine the antioxidant potential of DM extract. DM extract showed a better scavenging activity with an IC50 value of 90 ± 1.321055701. Ascorbic acid was used as a standard in DPPH assay showing an IC50 value of 65 ± 1.89.

### 3.4 DM extract promoted the metabolic activity of ADMSCs

**Figure 2.**
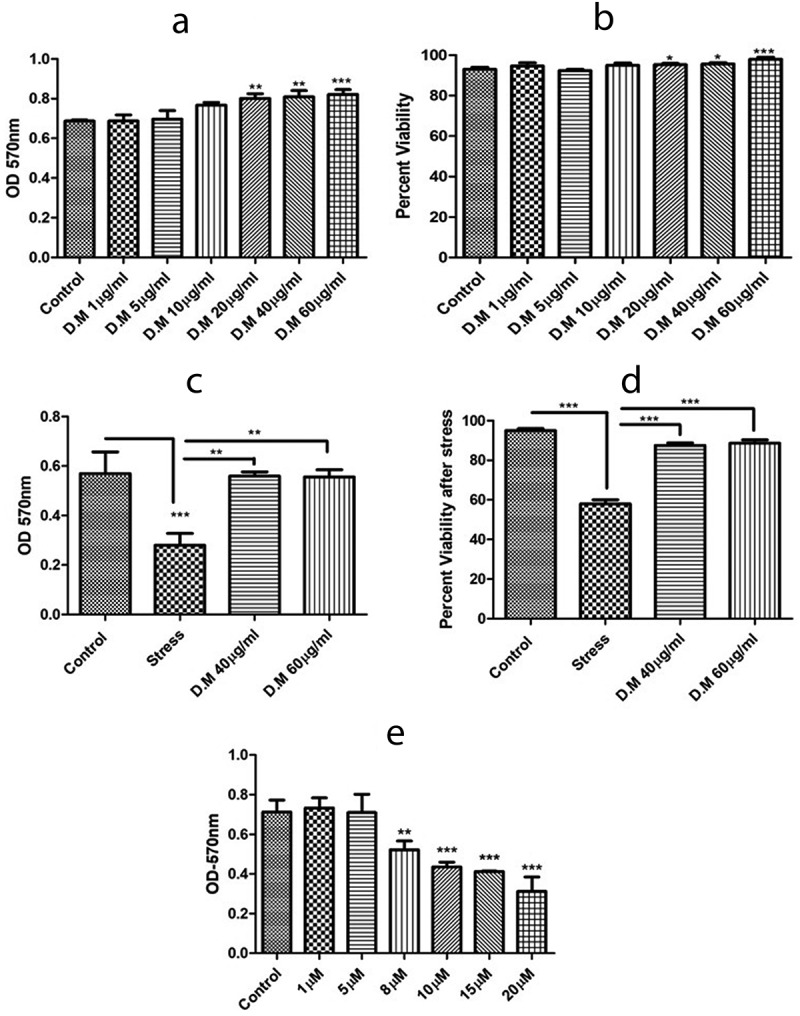
Metabolic activity and viability of hADMSCs in response to MIA stress and DM preconditioning. A. Metabolic activity of hADMSCs preconditioned with different doses of DM extract. B. hADMSCs viability against different DM concentrations. C. Metabolic activity of hADMSC’s preconditioned with DM extract concentrations followed by MIA induced oxidative stress. D. Percent viability of hADMSC’s after DM preconditioning and MIA induced oxidative stress. Values are statistically significant at p*<0.05, p*< 0.01 compared with the stress group. E. Graph representing metabolic activity response of hADMSC’s against monosodium iodoactate induced oxidative stress. The experiment was carried out in triplicates (n = 3)

DM extract showed an increase in metabolic activity with increase in DM concentration. High metabolic activity of DM extract was observed at 60 µg/ml concentration (Figure 2a). This suggests that DM increases the proliferative potential of hADMSCs.

### 3.5 DM extract improved viability of ADMSCs in stress condition

Tryphan blue exclusion assay confirmed the viability as high viability was observed at concentrations of 1 µg/ml and 60 µg/ml (Figure 2b) that indicated the protective potential of DM extract and suitability for preconditioning approach.

### 3.6 Monosodium iodoacetate caused cell death in a dose dependent manner

Monosodium iodoacetate stress at different concentrations reveals a decrease in cell viability with an increase in MIA concentration (Figure 2e). This clearly indicates that MIA induces stress in hADMSCs. We chose an optimum MIA stress of 10 µg/ml against hADMSCs as it induced stress but did not result in complete cell death.

### 3.7 Preconditioning with DM extract increased metabolic activity of stressed hADMSCs

For the assessment of metabolic activity, cells were preconditioned with two selected DM concentrations (40 µg/ml, 60 µg/ml) against MIA induced oxidative stress. Results affirmed that cells preconditioned with DM extracts at 40 µg/ml and 60 µg/ml showed significant metabolic activity as compared to stress (Figure 2c).

### 3.8 DM extract preconditioning improved viability of stressed hADMSCs

Cultured hADMSCs preconditioned with DM concentrations (40 µg/ml, 60 µg/ml) exhibited high viability in comparison to hADMSCs treated with MIA only (Figure 2d). Results proposed that DM extract preconditioned cells enhanced the protective ability of hADMSCs to cope with oxidative stress *in vitro* and hence increased their survival in harsh hypoxic microenvironment.

### 3.9 DM extract promoted cell migration

**Figure 3.**
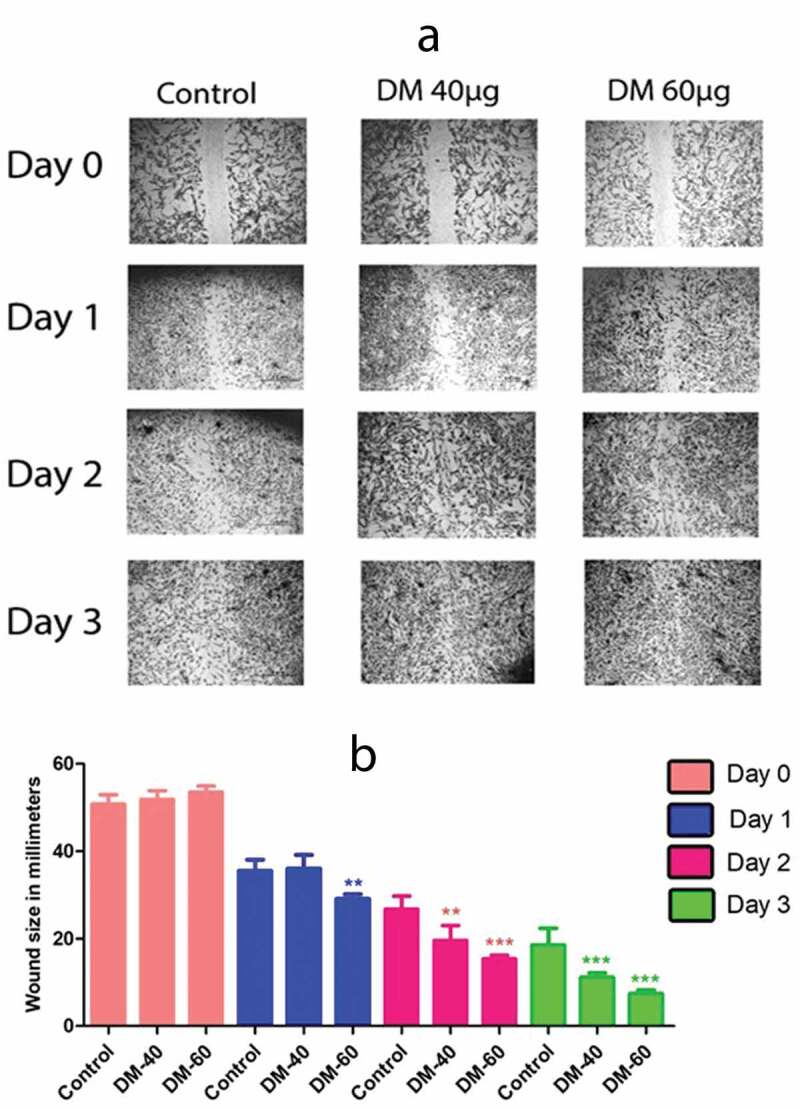
Migration potential of hADMSCs where (a) represents DM Preconditioned hADMSCs at concentrations of DM 40 µg/ml and DM 60 µg/ml shows an increased wound closure potential as compared to control group while (b) represents the statistical analysis of wound closure at day 0, day1, day2 and day 3 performed through ‘imagej’ software. Wound closure was evaluated as area covered by cells in millimetre (mm) of scratched area. DM-40 and DM 60 represents doses at 40 µg/ml and 60 µg/ml respectively

Decreased cell migration ability and hence less wound closure was observed in control group as compared to that of DM40µg/ml and DM 60 µg/ml preconditioned hADMSCs after Day 3. We observed an overall 32.277% wound closure in control at day3 while significant wound closure of 40.776% and 46.091% was observed in DM 40 µg/ml and 60 µg/ml respectively. Results affirm that cells preconditioned with DM extract exhibited a high migration and proliferative potential by showing a better wound closure (Figure 3a). Imagej analysis reveal no significance between control, DM40µg/ml and DM 60 µg/ml at day 0. However, a significant wound closure is evident at day 3 of DM40µg/ml and DM 60 µg/ml as compared to control group (Figure 3b).

### 3.10 DM preconditioning decreased cytotoxicity

**Figure 4.**
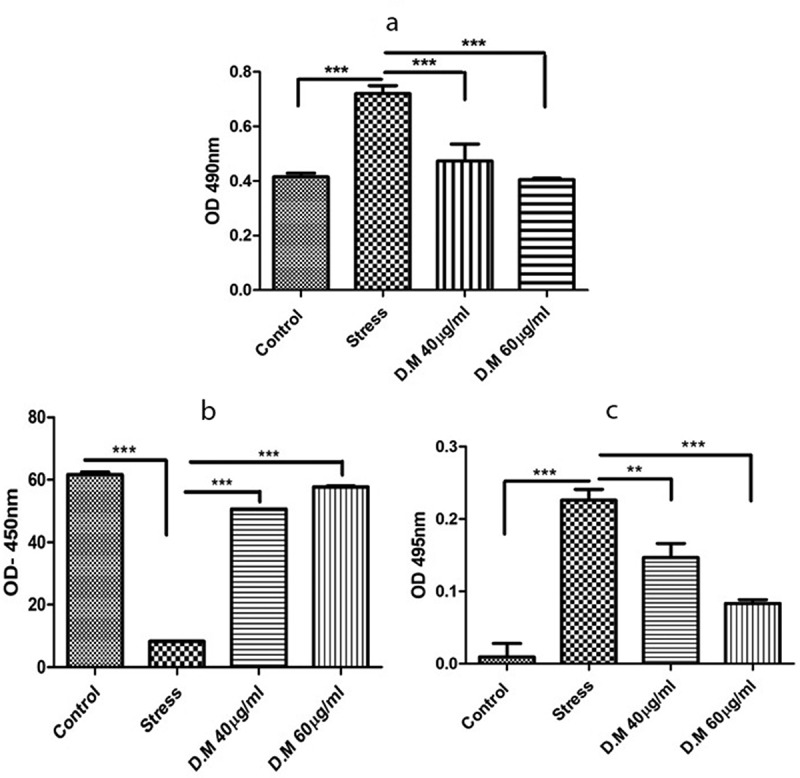
Biochemical assays of cells preconditioned with DM extract at 40 µg/ml and 60 µg/ml concentrations. LDH activity of DM preconditioned and MIA induced hADMSCs (a), Superoxide Dismutase activity of hADMSCs (b) and activity of reactive oxygen species within hADMSCS (c)

LDH release is directly proportional to damage of the plasma membrane. A high LDH release represents cell membrane damage/death. Herein, a significantly low LDH release was observed in MIA induced hADMSCs preconditioned with 40 µg/ml and 60 µg/ml DM concentrations as compared to stress control (Figure 4a).

### 3.11 DM preconditioning boosted antioxidant activity of ADMSCs

Superoxide Dismutase (SOD) is an important antioxidative enzyme present in the cells capable of reducing superoxide ions into less harmful products. SOD activity is high in normal cells as compared to cells experiencing oxidative stress. We observed a high SOD activity in DM preconditioned hADMSCs as compared to MIA induced hADMSCs (Figure 4b).

### 3.12 DM preconditioned increased reactive oxygen species scavenging capacity

Results reveal that cells preconditioned with 40 µg/ml and 60 µg/ml DM concentrations assisted in decreasing reactive oxygen species in MIA induced hADMSCs as compared to stress (Figure 4c).

### 3.13 DM preconditioning down-regulated the pro-inflammatory markers

**Figure 5.**
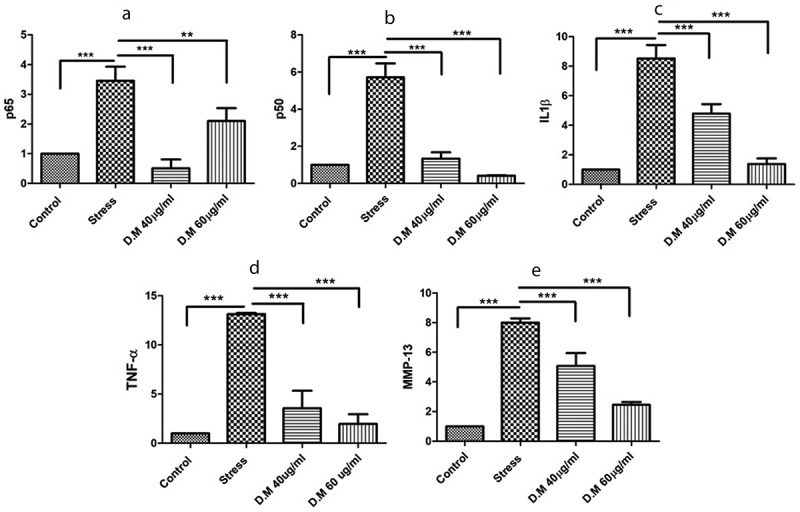
The mRNA expression level of proinflammatory markers where, (a) represents expression levels of TNF-α, (b) indicates mRNA levels of p65, (c) reveals the expression levels of p50, (d) fold change of MMP-13 and (e) indicates the expression level of IL1β

DM preconditioned cells exposed to MIA stress revealed significant down regulation of **p65** (MIA-10uM: 3.457402989 ± 0.475844547, **DM**-40 µg/ml: 0.50875056 ± 0.297812567 and **DM**-60 µg/ml: 2.103011986 ± 0.432728407), **p50** (MIA-10uM: 5.717260329 ± 0.745198225, **DM-**40 µg/ml: 1.336867298 ± 0.339661368 and **DM-**60 µg/ml: 0.414975647 ± 0.0196445), **IL1β** (MIA-10uM: 8.526609879 ± 0.912115066, **DM-**40 µg/ml: 4.798220188 ± 0.642921904 and **DM-**60 µg/ml: 1.387847877 ± 0.371164421), **TNF-α** (MIA-10uM: 13.147330 ± 0.1054702, **DM-** 40 µg/ml: 3.568434 ± 1.777000 and **DM-**60 µg/ml: 1.971887 ± 0.9693008) and **MMP-13** (MIA-10uM: 8.003435655 ± 0.285201825, **DM-** 40 µg/ml: 5.080665945 ± 0.863096022 and **DM-** 60 µg/ml: 2.46127352 ± 0.182866937) as depicted in (Figure 5) . Values represented are statistically significant at p*<0.05, p*< 0.01 compared to the stress group.

### 3.14 DM affected the paracrine release of ADMSCs

ELISA results depicted the significant decrease in **(A)** TNF-α (MIA-10uM: 37.571430 ± 4.237483, DM-40 µg/ml: 14.897440 ± 0.3925792 and DM-60 µg/ml: 13.038460 ± 0.7925144), **(B)** p65 (MIA-10uM: 0.562000 ± 0.022000, DM- 40 µg/ml: 0.369500 ± 0.022500 and DM-60 µg/ml: 0.310500 ± 0.009500), **(C)** IL1β (MIA-10uM: 37.571430 ± 4.237483, DM-40 µg/ml: 11.619050 ± 2.462071 and DM-60 µg/ml: 12.571430 ± 0.7901638) and **(D)** IKKα and IKKβ (MIA-10uM: 0.764000 ± 0.01555635, DM-40 µg/ml: 0.151000 ± 0.0888313 and DM-60 µg/ml: 0.082500 ± 0.01664332) protein concentrations in DM preconditioned and stressed hADMSCs. Represented values are statistically significant at p*<0.05, p*< 0.01 compared with the stress group.

**Figure 6.**
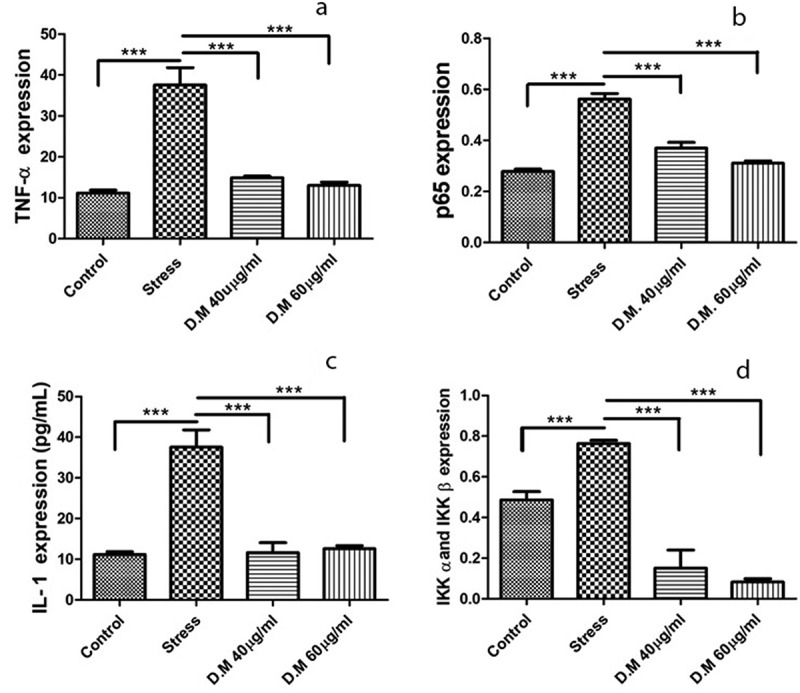
Cytokine concentrations (n = 3) of control, stress and DM preconditioned groups where (a) represents protein concentration of TNF-α (b) represents protein concentration of p65 while (c) indicates the protein concentrations of IL1β while (d) represents the protein concentrations IKK-α and IKK-β

## 4. Discussion

The heterogeneity of isolated cells hinders the MSC-based therapies [45]. Besides plasticity and multipotency, the MSCs are positive for CD73, CD90 and CD105 while negative for CD11b or CD14, CD19 orCD79a, CD34, CD45, and HLA-DR in their cell-surfaceimmunophenotype [11].Another study also reports the positive expression of CD49d while negative expression of CD106 [46]. Herein, isolated MSCs exhibited positive expression for CD73, CD90, CD105 and CD49d while negative expression of CD34, CD45 and CD106 cell surface markers (Figure 1). MSCs are of great interest clinically as they are believed to replace cells lost due to ageing or tissue injury [11]. However, one of the disadvantage of MSCs is their less survival capability in stress conditions [18]. Various conditions including harsh hypoxic microenvironment and inflammation play a vital role in less survival of MSCs [47,48]. To overcome this problem, MSCs are preconditioned with different compounds including phytochemicals [19,20]. Several studies report that preconditioned MSCs have a great survival, proliferation and as well as therapeutic potential because they can better cope with the inflammatory conditions both *in vitro* and *in vivo* as compared to the non-preconditioned MSCs [26].

Chemical compounds from plants including steroids, terpenoids, sterols, flavonoids, sesquiterpene lactones, curcuminoids, thymoquinones, triterpenes, phytoalexins were proven to exhibit antioxidant, anti-inflammatory activity against several diseases [49]. Phenolic compounds, in particular, show a high reactive oxygen savaging capacity and hence exhibits greater antioxidant activity [50]. *Daphne mucronata* is traditionally used in South Asia to treat various inflammatory disorders [37]. Our phytochemical investigation of DM extract reveals the presence of high phenolic compounds thus exhibiting better reactive oxygen scavenging capacity when subjected to DPPH assay.

Despite the traditional use of *Daphne mucronata* against several disorders, its protective and proliferative potential in MSCs has not been investigated yet. The isolated hADMSCs exhibit the same morphological features as previously reported [51] Herein, we have assessed the metabolic activity and viability of *Daphne mucronata* against hADMSCs. We observed a 17.8% and 21% increase in metabolic activity of DM treated MSCs at 40 µg/ml and 60 µg/ml concentrations respectively (Figure 2a). Moreover, a 100% viability in hADMSCs were observed revealing no toxic effect of DM on hADMSCs at 40 µg/ml and 60 µg/ml concentrations (Figure 2b). Previous studies demonstrate a high metabolic activity and viability outcome in plant extract treated cells [52,53]. Similarly, the scratch assay is an *in vitro* model that mimics *in vivo* incisional wound model [54]. Cell migration assay is extensively used to evaluate the migration of cells and proliferative potential of plant extracts on various cells [55,56]. The present study, in correspondence to the previous studies, reports that hADMSCs treated with DM extract exhibited a high migration potential to that of untreated (Control) cells (Figure 3).

Furthermore, oxidative stress was induced in hADMSCs using MIA to assess the protective potential of DM extract. DM preconditioned MIA induced hADMSCs were able to withstand hypoxic conditions exhibiting high metabolic activity and viability as compared to MIA induced hADMSCs (Figure 2c,D). Our results are in correspondence with the study demonstrating the protective and proliferative effect of *curcumin* extracts against hADMSCs [57].

Additionally, high LDH release has been reported in damaged cells as compared to normal cells [58,59]. The decreased level of LDH activity in DM preconditioned groups suggests low cellular damage against MIA stress (Figure 3a). Similarly, oxidative stress results due to reactive oxygen species that damage DNA and ultimately results in cell apoptosis [60]. Herein, the low ROS activity indicates the less reactive oxygen species presence within the cells. Conversely, superoxide dismutase is an antioxidant enzyme present in the cells that catalyzes the conversion of superoxide anion into less toxic hydrogen peroxide and molecular oxygen [61]. The increase in SOD activity is also related to the decrease in oxidative stress to the cells [62]. A high SOD activity clearly indicates the positive regulation of superoxide dismutase enzyme by cellular machinery to cope with the oxidative stress within the cells.

To mimic oxidative stress and inflammatory conditions *in vitro*, we used MIA to induced stress conditions (Figure 2e) as MIA is used to induce oxidative stress that further leads to upregulation of various proinflammatory pathways [34]. When it comes to inflammation process, it is well known that different signalling pathways are involved that impair homoeostasis [63]. The nuclear factor NF-κB pathway has long been considered a prototypical proinflammatory signalling pathway, largely based on the role of NF-κB in the expression of proinflammatory genes including cytokines, chemokines, and adhesion molecules [64]. The NF-κB family consists of five members: RelA (p65), RelB, c-Rel, NF-κB1 (p50 and its precursor p105), and NF-κB2 (p52 and its precursor p100) [65]. The most prevalent activated form is the heterodimer RelA (p65) and p50 [66]. We assessed the expression level of p65 and p50 markers. A significant decrease in the mRNA expression of both p65 and p50 markers was observed in DM preconditioned hADMSCs groups as compared to MIA induced hADMSCs (Figure 5a,b). The p65 marker was also analysed further at protein level through ELISA that revealed a significant decrease in DM preconditioned groups (Figure 6b). These results are consistent with the previous reports [67,68]. Similarly, IKKα and IKKβ also serves as a core element for the regulation of NF-κB pathway [69]. (inhibitor of kappa kinase α (IKK-α) and (inhibitor of kappa kinase β (IKK-β) are prominent regulators of NF-κB (non-canonical) pathway [70]. The IKK-α and IKK-β phosphorylates the IκB protein that binds the p65 and p50 heterodimer thereby releasing the heterodimer resulting in regulation of NFκB pathway [71]. The current study indicates a significant decrease in the IKK-α and IKK-β protein levels resulting in downregulation of proinflammatory markers that are positively regulated through NFκB pathway suggesting the inhibitory effect of DM extracts against these markers. (Figure 6d). The down regulation of IKK-α and IKK-β has been reported in LPS-stimulated RAW 264.7 cells using *Populus deltoides* leaf extract [72]. Additionally, NF-κB positively regulates genes encoding cytokines including TNF-α and IL-1β [73]. It has been reported that both these cytokines are directly involved in inflammation [74]. To assess the gene regulation of these cytokines, the expression level of TNF-α and IL1-β markers were also evaluated. Our results reveal a decrease in mRNA (Figure 5c,d) and protein levels (Figure 6a,c) of TNF-α and IL1β markers in DM preconditioned hADMSCs. Similar down regulation at mRNA and protein level of IL1β beta and TNF-α has recently been reported in Lipopolysaccharide-Induced Human Monocyte-Derived Macrophages [75]. On the other hand, matrix metalloproteinase-13 (MMP-13) is a metalloproteinase enzyme that is involved in the degradation of extracellular matrix [76,77]. Increased MMPs expression has been noticed in inflammatory conditions [78]. Despite of their role in differentiation process, MMP-13 helps in activation of TNF proinflammatory marker by cleaving pro-TNF into bioactive TNF [79]. Previous studies also reports the activation of IL1β by MMPs through proteolytic processing [80]. Here we observed a high MMP-13 expression in MIA induced hADMSCs in comparison to DM preconditioned hADMSCs (Figure 5e). Prior studies conducted also indicates downregulation of MMP-13 has been reported in human HNSCC, HN22, HSC-3 and RAW 264.7 cell lines using *Tinospora crispa* and *Morus alba* plant extracts [81,82].

## 5. Conclusion

Thus this research reports the anti-inflammatory, antioxidant and cell proliferative ability of *Daphne mucronata* preconditioned hADMSCs. In conclusion, DM preconditioning of hADMSCs ameliorated the adverse effects induced *in vitro* through MIA stress by activating the NF-κB pathway. The result can contribute to the therapeutic applications of DM preconditioned hADMSCs for the treatment of inflammatory diseases.
